# Micronucleus frequency in buccal mucosa cells of patients with neurodegenerative diseases

**DOI:** 10.1038/s41598-020-78832-y

**Published:** 2020-12-17

**Authors:** Hauke Reimann, Helga Stopper, Thomas Polak, Martin Lauer, Martin J. Herrmann, Jürgen Deckert, Henning Hintzsche

**Affiliations:** 1 Institute of Pharmacology and Toxicology, University of Würzburg, Versbacher Straße 9, 97078 Würzburg, Germany; 2 Department of Psychiatry, Psychosomatics and Psychotherapy, Center of Mental Health, University Hospital Würzburg, Würzburg, Germany; 3 Bavarian Health and Food Safety Authority, Erlangen, Germany

**Keywords:** Genetic markers, Chromosome abnormality, Alzheimer's disease

## Abstract

Neurodegenerative diseases show an increase in prevalence and incidence, with the most prominent example being Alzheimer’s disease. DNA damage has been suggested to play a role in the pathogenesis, but the exact mechanisms remain elusive. We enrolled 425 participants with and without neurodegenerative diseases and analyzed DNA damage in the form of micronuclei in buccal mucosa samples. In addition, other parameters such as binucleated cells, karyolytic cells, and karyorrhectic cells were quantified. No relevant differences in DNA damage and cytotoxicity markers were observed in patients compared to healthy participants. Furthermore, other parameters such as lifestyle factors and diseases were also investigated. Overall, this study could not identify a direct link between changes in buccal cells and neurogenerative diseases, but highlights the influence of lifestyle factors and diseases on the human buccal cytome.

## Introduction

Dementia is one of the most common neurodegenerative disorders. The most frequent disease associated with dementia is Alzheimer’s disease (AD), which can currently not be cured^1^. In 2015, 47 million people worldwide were affected, a number which is predicted to increase to 132 million in 2050^2^. The global costs of dementia were estimated to be $818 billion in 2015, which is an increase of 35.4% compared to 2010 and represented 1.09% of the world gross domestic product^3^.

Early symptoms of AD include memory loss, other cognitive impairments and abnormal behaviour, whereas characteristic cellular pathologic signs of AD are the presence of extracellular amyloid plaques and neurofibrillary tangles^4^. The definitive diagnosis of AD is only possible post mortem via histopathology, therefore only a clinical diagnosis based on symptoms is currently possible. For this, a structured patient history of any cognitive and functional abnormalities along with a neurological examination is necessary^5^. One of the most prominent tests used for detection of AD, as well as for its pre-stage mild cognitive impairment (MCI), is the Mini-Mental State Examination (MMSE), which can be performed easily and quickly. A score below 27 of 30 in the MMSE indicates cognitive impairment, but this does not allow a clear distinction between AD and MCI. Unfortunately, the MMSE is not very sensitive or specific and especially the true diagnosis of early forms of AD and MCI remains problematic^6^. As the treatment of AD is more successful, if only few neurons are affected, an early detection of AD/MCI would be crucial for therapy success and patient welfare^7^.

To overcome this issue, further diagnostic tools are needed and are currently under development, including biomarkers to predict and investigate AD or MCI^8,9^. Important criteria for the ideal biomarker are, amongst others, reproducibility, feasibility, cost, sensitivity and specificity^10^. One biomarker of interest is DNA damage, not only in the context of diagnosis but also for better understanding the underlying pathophysiological mechanisms^9^. Many neurological dysfunctions like AD are associated with DNA damage^11^. An important factor for the aetiology of AD is thought to be oxidative stress, which is often increased before any other symptom of a disease occurs and is related to important steps of AD development like amyloid-β plaques and neurofibrillary tangles^12^. There are diverse effects of oxidative stress in cells but DNA damage is one of the most prominent consequences of elevated oxidative stress levels^13^. Therefore, the investigation of various types of DNA damage as biomarker like base damage (e.g. 8-OHdG), telomere integrity and chromosomal damage in the form of micronuclei (MN) is highly relevant^9,14^. MN can result from chromosomal fragments after DNA strand breaks or whole chromosomes, which are not divided to one of the spindle poles during cell division^15^. The in vitro MN test is a frequently used and standardised test for the determination of genomic instability^16^. The test can be performed in various cell types, including human buccal cells. When cell death parameters and regenerative potential are analysed in addition to DNA damage, the test is called buccal MN cytome assay^17^. Apart from MN, also cells undergoing cell death or showing errors in cell division can be quantified^18^. Buccal cells have some properties that make them suitable as biomarkers, particularly they can be collected non-invasively and the preparation and evaluation of the buccal cells is cheap and does not need any high-tech equipment^17^. The MN test in buccal cells has been used to investigate effects after local exposure of genotoxins or oral cancer forms, but has furthermore been used to study the chromosomal instability from other diseases or lifestyle factors^19,20^.

In this study, we performed the buccal MN cytome test in a large group of elderly people with and without neurodegenerative diseases in order to better understand the link between genomic instability and neurological disorders.

## Results

Out of 329 individuals of the reference group without severe neurological disease, 43 had a diagnosis of MCI and 32 of depression at the time of sampling. Only a minority of 14 of 327 of the reference group with a record of MMSE had a score below 27. Gender was equally distributed (168 females and 161 males). The same is true for the patient group (51 female and 44 male patients respectively), but the number of individuals with MMSE below 27 was increased compared to the reference group (43 of 78 individuals with known MMSE). Detailed information on both groups are provided in Tables 1 and 2.

**Table 1:** Characterisation of recruited reference group with available information at time of sampling.

	Reference		
Number	329		
Mean age	76.33		
Gender	Female 168	Male 161	
BMI	Normal 133	Pre-adipose 142	Adipose 49
Smoking	Non-smoker 251	Smoker 77	
Alcohol	No alcohol 214	Alcohol consumption 113	
Mental state	Healthy 249	MCI 43	Depression 32
Cancer	No cancer 281	Cancer 44	
MMSE	MMSE ≥ 27 314	MMSE < 27 14

**Table 2:** Characterisation of patients with available information at time of sampling.

Patients					
Number	Mean age	Gender		MMSE	
		Female	Male	MMSE ≥ 27	MMSE < 27
96	73.24	51	44	35	43

### Comparison of reference group and patient group

**Figure 1:**
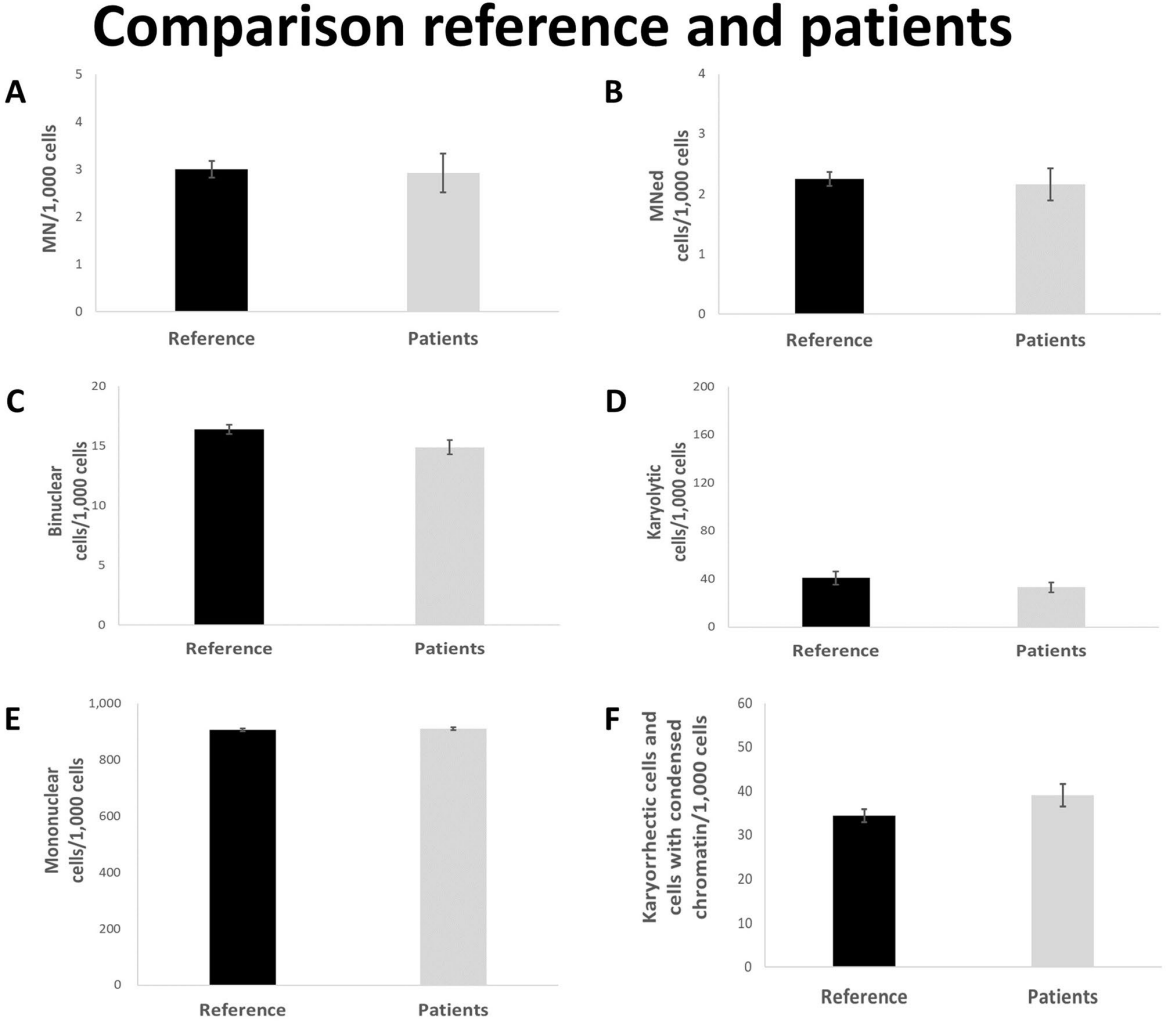
Rate of MN (**A**), MNed cells (**B**), binucleated cells (**C**), karyolytic (**D**), mononuclear (**E**) and karyorrhectic cells and cells with condensed chromatin (**F**) per 1000 cells. Mean of each group ± standard error. Black bars represent data from reference, grey bars represent data from patients. n (reference): 329; n (patients): 96.

Both cohorts (reference and patient groups) show similar results regarding the parameters MN, micronucleated (MNed) cells, karyolytic cells and mononuclear cells (Fig. 1a,b,d,e). Only small differences with regard to the frequency of binucleated cells (Fig. 1c, reduced in patients), and karyorrhectic and condensed chromatin cells (Fig. 1f, elevated in patients), can be seen.

The same can be observed, when individuals are stratified by gender (Supplementary Fig. 1), with the exception of a very small but statistically significant difference between reference and patient group in the number of mononucleated cells in females (Supplementary Fig. 1e). Beside this, there were no clear differences attributable to gender.

**Figure 2:**
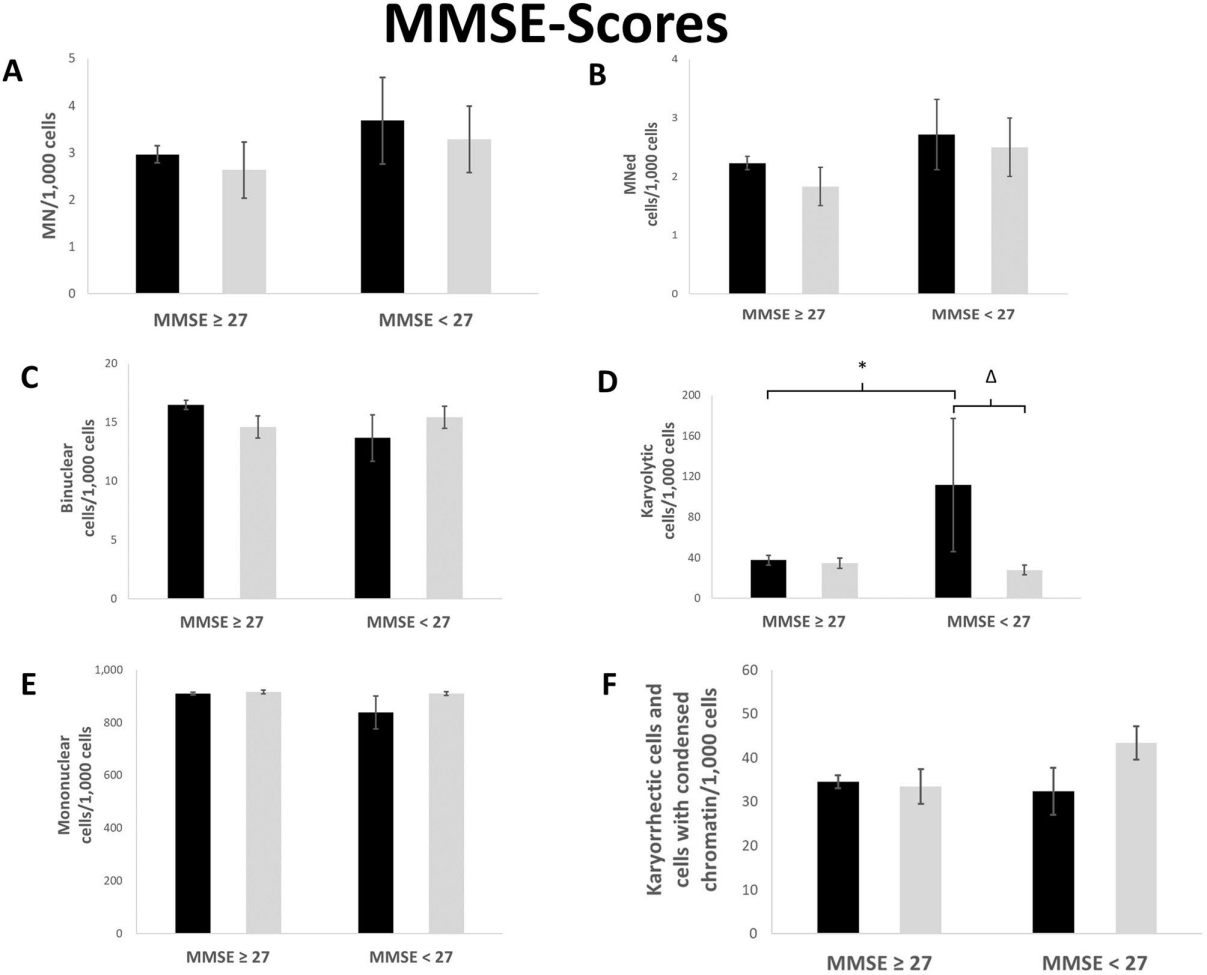
Rate of MN (**A**), MNed cells (**B**), binucleated cells (**C**), karyolytic (**D**), mononuclear (**E**) and karyorrhectic cells and cells with condensed chromatin (**F**) per 1000 cells. Mean of each group ± standard error. Black bars represent data from reference, grey bars represent data from patients. n (MMSE ≥ 27 reference): 314; n (MMSE < 27 reference): 14. n (MMSE ≥ 27 patients): 35; n (MMSE < 27 patients): 43. Asterisk represent *p* value < 0.05 after statistical evaluation referring to MMSE ≥ 27. Delta represent *p* value < 0.05 after statistical evaluation referring to reference.

Differences were observed when reference and patient groups were stratified according to MMSE score with a threshold of 27. However, these differences did not reach significance. MN and MNed cells were elevated in samples from reference or patient groups with a MMSE lower than 27 compared to individuals with a MMSE of 27 or higher (Fig. 2a,b). Reference group members with a MMSE below 27 also showed an elevated rate of binucleated cells compared to reference group members with a MMSE of 27 or higher, whereas in the patient group there was no clear difference (Fig. 2c). A significant increase could be furthermore be seen in karyolytic cells from reference group members with MMSE < 27 compared to those with a MMSE of 27 or higher, which could not be detected in patient group members with a similar MMSE score (Fig. 2d). Only a slight increase of mononuclear cells was observed in patient group members with MMSE < 27 compared to reference group (Fig. 2e). On the other hand, karyorrhectic and condensed chromatin cells were elevated in patient group members with high MMSE score compared to those with a low score or with reference group members (Fig. 2f).

### Impact of possible lifestyle factors and disease status

In addition, various potential confounding factors like weight, smoking, alcohol consumption, cancer diagnosis or mental state (depression) on the buccal cell cytome of reference group were investigated. None of these parameters showed any significant difference between females and males (Supplementary Fig. 1). In males, a slight tendency for less MN and MNed cells (Supplementary Fig. 1a,b) and more karyolytic, karyorrhectic and condensed chromatin cells (Supplementary Fig. 1d,f) could be observed.

A non-significant increase of MN and MNed cells was observed in buccal cells of adipose individuals compared to those with normal BMI value of 20–25 (Supplementary Fig. 2a,b). No differences in BMI could be seen when bi-, mononuclear or karyorrhectic/condensed chromatin cells were considered (Supplementary Fig. 2c,e,f). In addition, there was a significant increase in the number of karyolytic cells of pre-adipose (BMI: 26–30) and adipose individuals (BMI > 30, Supplementary Fig. 2d).

**Figure 3:**
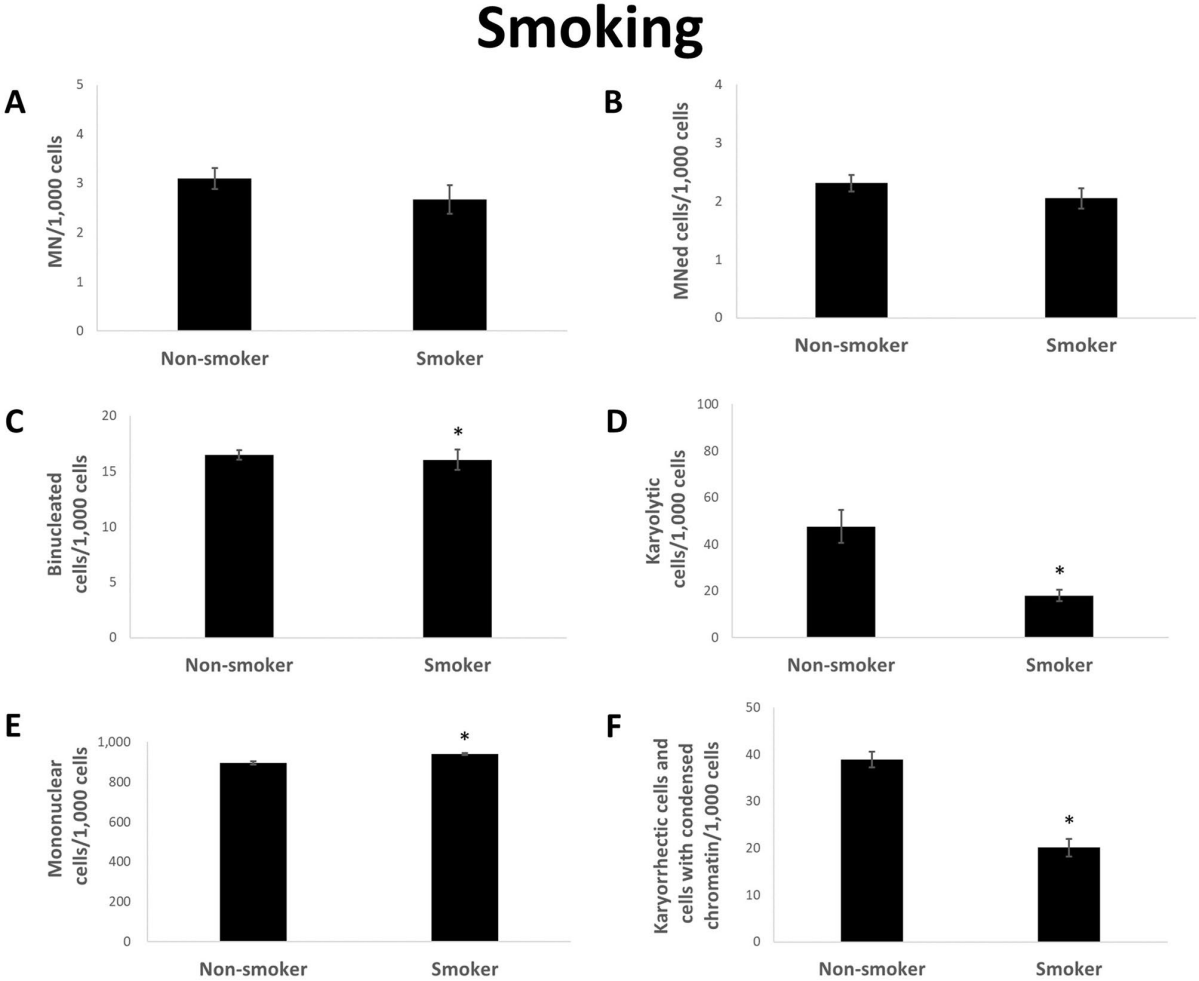
Rate of MN (**A**), MNed cells (**B**), binucleated cells (**C**), karyolytic (**D**), mononuclear (**E**) and karyorrhectic cells and cells with condensed chromatin (**F**) per 1000 cells from reference distributed by smoking habit. Mean of each group ± standard error. n (non-smoker): 251; n (smoker): 77. Asterisk represent *p* value < 0.05 after statistical evaluation referring to non-smoker group.

When smoking habit is considered, no significant increase in the number of MN and MNed cells could be observed (Fig. 3a,b). In contrast, even a small decrease in both parameters could be seen. In addition, a significant decrease in the number of karyolytic, karyorrhectic and condensed chromatin cells was observed when smokers were compared to non-smokers (Fig. 3d,f). Statistically significant but small changes occurred at bi- and mononuclear cells in smokers (Fig. 3c,e).

**Figure 4:**
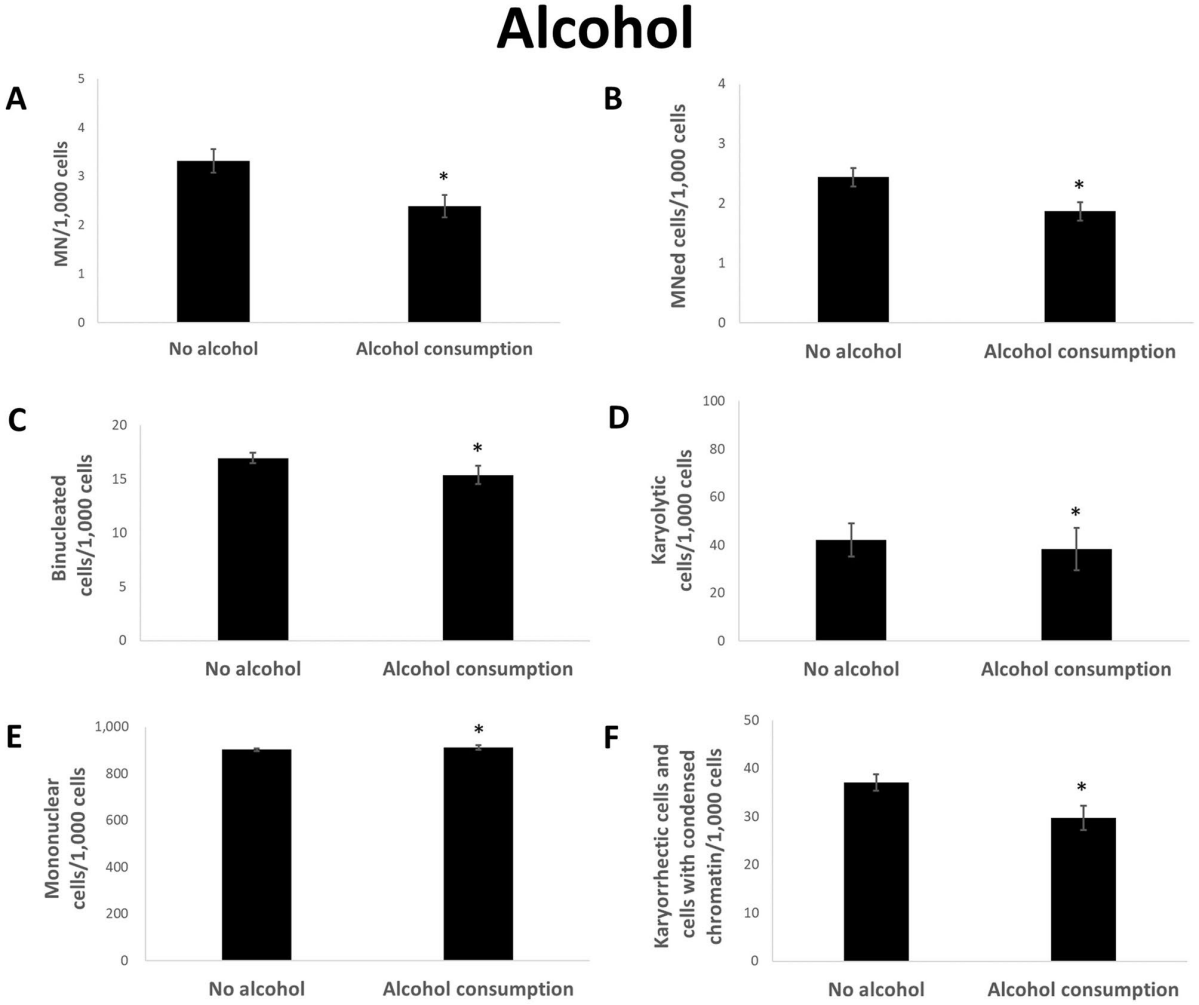
Rate of MN (**A**), Mned cells (**B**), binucleated cells (**C**), karyolytic (**D**), mononuclear (**E**) and karyorrhectic cells and cells with condensed chromatin (**F**) per 1000 cells from reference distributed by alcohol consumption. Mean of each group ± standard error. n (no-alcohol): 214; n (alcohol consumption): 113. Asterisk represent *p* value < 0.05 after statistical evaluation referring to non-alcohol consuming group.

The consumption of alcohol lead to a significant reduction in the number of MN and all other parameters (Fig. 4a,b,c,d,f), the only exception being mononucleated cells, which were increased in the group of alcohol consumers (Fig. 4e).

**Figure 5:**
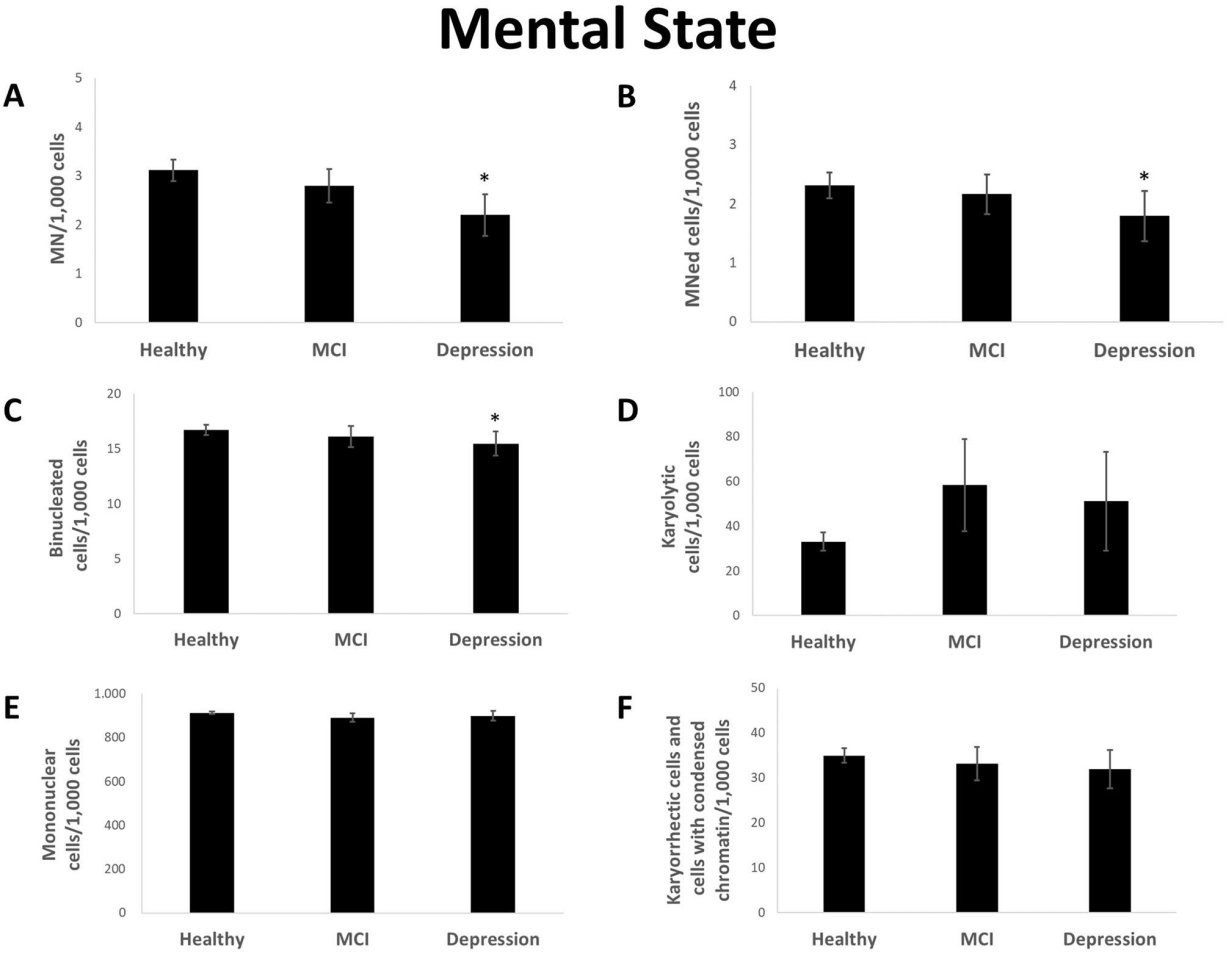
Rate of MN (**A**), MNed cells (**B**), binucleated cells (**C**), karyolytic (**D**), mononuclear (**E**) and karyorrhectic cells and cells with condensed chromatin (**F**) per 1000 cells from reference distributed by mental state. Mean of each group ± standard error. n (healthy): 249; n (MCI): 43; n (depression): 32. Asterisk represent *p* value < 0.05 after statistical evaluation referring to reference.

Significantly less MN and MNed cells were detected in otherwise healthy individuals suffering from depression (Fig. 5a,b). In addition, these parameters showed no or only a very slight decrease in persons with MCI (Fig. 5a,b). A slight reduction in binucleated cells was found in individuals with MCI and depression (Fig. 5c). Furthermore, the rate of karyolytic cells was increased in these groups (Fig. 5d). In contrast, karyorrhectic/condensed chromatin cells and mononuclear cells showed no changes in participants with MCI or depression (Fig. 5e,f).

**Figure 6:**
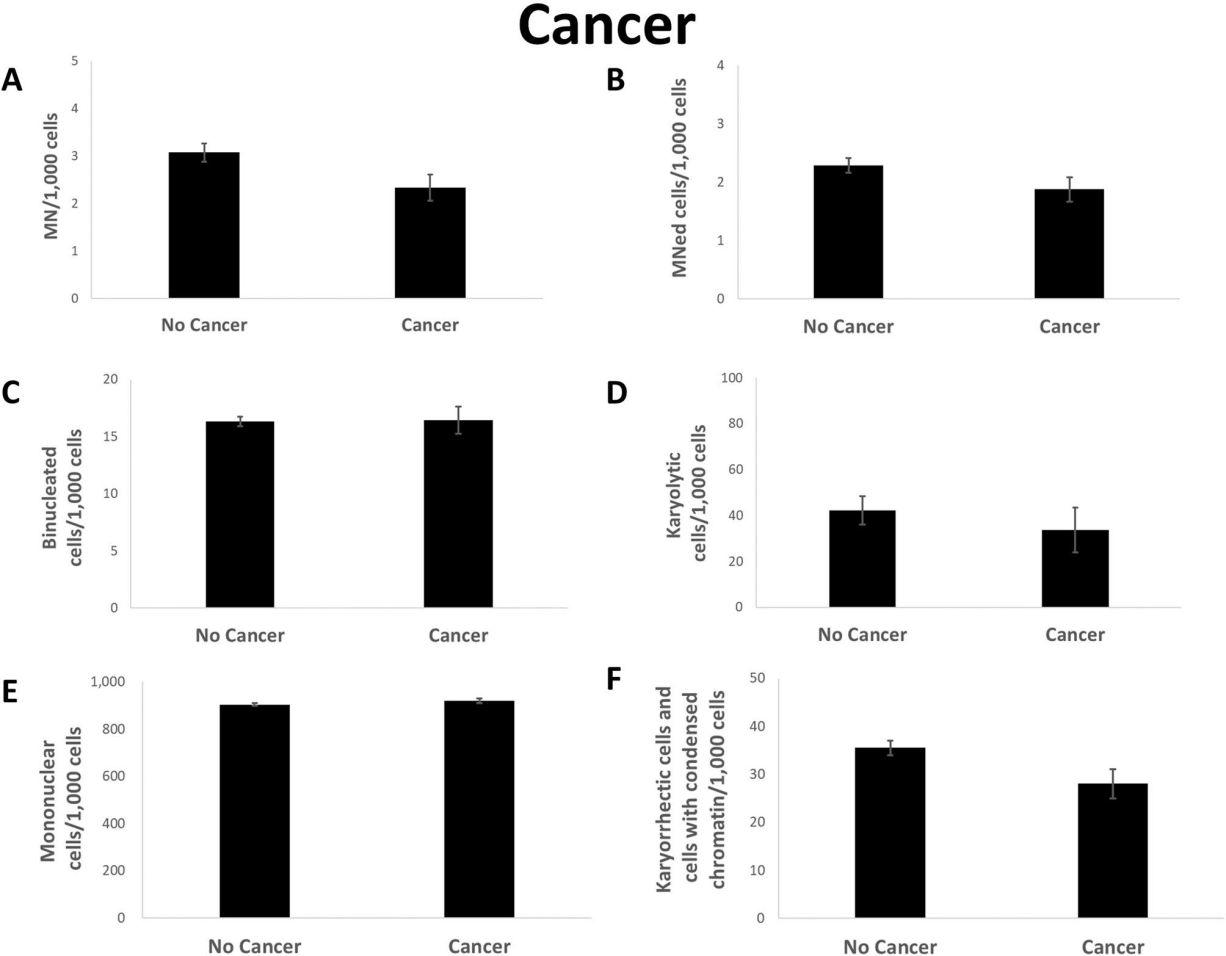
Rate of MN (**A**), MNed cells (**B**), binucleated cells (**C**), karyolytic (**D**), mononuclear (**E**) and karyorrhectic cells and cells with condensed chromatin (**F**) per 1000 cells from reference distributed by cancer occurrence. Mean of each group ± standard error. n (no cancer): 281; n (cancer): 44. Asterisk represent *p* value < 0.05 after statistical evaluation referring to healthy individuals.

Interestingly, less MN and Mned cells were observed in buccal cells of cancer patients (Fig. 6a,b). Reference and patients with cancer both suffered from a large variety of different cancer forms. Less karyorrhectic and condensed chromatin cells but slightly more binucleated cells could be seen (Fig. 6c,f). No changes in mononuclear cells between cancer and non-cancer group was noticed (Fig. 6e). Furthermore, the number of karyolytic cells was reduced in people suffering from cancer (Fig. 6d).

## Discussion

MN result either from chromosomal fragments, which indicate DNA strand breaks or chromosome missegregation^15^ and are associated with a variety of neurological diseases^21^. We performed the buccal cell MN cytome test in a large cohort to compare a reference group without severe neuropsychiatric disorders with patients suffering from such a disease at the time of sampling. In the present study, MN were not only counted in mononuclear cells, but also in cells with condensed chromatin and in binuclear cells, which could lead to slightly higher MN frequencies compared to other studies, although the amount of MN scored in binuclear cells or cells with condensed chromatin is very small compared to mononuclear cells. Nevertheless, the magnitude of the MN frequencies is roughly in the range that is usually reported, showing good comparability with other studies. Another explorative aim of the study was to further investigate the influence of different lifestyle factors and diseases on the outcome of the test. In total, over 400 samples were evaluated to reduce the effect of outliers and artefacts. A similar age cohort was chosen (mean age 76 vs 73), because age may pose a strong influencing factor e.g. MN frequency is increased by age in buccal cells, which could explain the comparably high number of observed MN in this study^20^.

None of the examined parameters showed any significant difference, when only the reference group was compared to the patient group. According to the present data, gender is no factor with clear effects on the buccal cytome. Furthermore, an increase of cytotoxicity parameters like karyolytic cells was observed in the subgroup with an MMSE < 27, therefore reduced mental capacity could be related to an increased rate of cell death. In addition, a slight, non-significant increase in the rate of MN and MNed cells were observed in both investigated groups for individuals with MMSE < 27, indicating a possible link between reduced mental capacity and increased MN frequency. Another study found a negative correlation of MMSE and MN frequency in lymphocytes in AD patients but not for individuals with MCI^22^. A partly different result was obtained in a study, where buccal cells from young and old AD patients were compared to control samples^23^. A slight and non-significant increase in Mned cells was only observed in young AD patients, but older AD patients had less MNed cells and both groups had significantly less cells indicating cell death (karyorrhectic, condensed chromatin and karyolytic cells). One explanation could be alterations in the level of vitamin B_12_ and homocysteine, which might impact the frequency of some investigated parameters^24^. In a cytokinesis-block cytome test, no significant differences between AD and control could be found but an increase in the frequency of nuclear buds between MCI cases and control^22^. An older study investigating MN in lymphocytes found a clear increase in MN in AD patients, which might indicate aneuploidy^25,26^. Both familial and sporadic AD caused an increase in MN frequency in lymphocytes in a further study^27^.

To investigate the possible interrelationship between the buccal cytome and mental alterations in more detail, the appearance of MCI in otherwise healthy (i.e. without severe disease such as dementia) reference group members was examined. Surprisingly, there was no increase in MN nor MNed cells during the time of sampling in individuals with MCI. An explanation of this finding might be derived from the aetiology of AD: oxidative stress is observed in early phases of AD and declines with disease duration, therefore chromosomal instability resulting from oxidative stress may be only visible during a limited time or can never be observed in buccal cells^12^. In contrast, another study found a strong positive correlation between MN frequency in lymphocytes of persons with MCI^28^. Also, for other diseases like Parkinson’s disease or Down syndrome an increase in MN frequency but also other DNA damage markers was found in various tissues^21^.

Taking together these findings, there seems to be no clear evidence, if AD increases DNA damage in the form of MN. Eventually, other confounding factors not considered in the studies above may have distorted the results. However, a direct comparison with our results is not possible, because an AD diagnosis is not identical to a MMSE score < 27 or MCI, for which only few information are available, that do not allow a conclusion. Parameters like karyolytic cells may pose a more sensitive marker for investigating genomic damage related to mental state.

MN were reduced in reference group with major depression. This is a contradicting result to the fact, that elevated oxidative stress levels and reduced DNA damage repair capacity were observed in people suffering from depression, which both should result in an increased DNA damage level^29^. Antioxidant enzymes can be induced by antidepressants, but it remains questionable, if this may have led to a higher antioxidant capacity of the individuals suffering from depression compared to the remaining reference group members or if it can only balance out the increased oxidative stress level in affected individuals^30^. Other mechanisms than oxidative stress have probably caused the decrease of this DNA damage marker. As also karyolytic cells were elevated in depressive patients, cell death pathways might be associated with the pathophysiology of depression. Although there has been some evidence for a long time that depression increases cell death rate in neuronal tissue, it is surprising that similar effects could be seen in buccal cells^31^.

Many other factors could influence the buccal cytome, like gender, life-style factors and diseases^20^. Only small non-significant differences could be found in our study for gender and BMI, but the (non-significantly) higher MN frequency in adipose reference group members is interesting, because other studies have found that obesity causes DNA damage and also buccal cells seem to be a sensitive marker for prediction of potential DNA damage from obesity^32,33^.

No increase in MN frequency could be found in smoking reference group individuals. Studies found, that only heavy smoking (≥ 40 cigarettes/day) causes a significant increase in MN in buccal cells^20^. Most of the smokers were only light smokers, which could explain the lack of a rise in MN. Similar to smoking, effects on the buccal cytome after alcohol consumption were found in other publications only at high doses, although they remained non-significant^20^. Interestingly, high alcohol doses caused a reduction of MN, which was also observed in this study and is contradictory to the expected toxicity of alcohol, especially at one of the most prominent tissues of exposure.

Cancer is generally associated with DNA damage, which makes the buccal cell MN cytome assay suitable as a sensitive marker for many cancer types^19^. Therefore, it is surprising that samples from reference group members with cancer show even slightly reduced amounts of MN and less karyolytic, karyorrhectic and condensed chromatin cells. A recent review on the effect of environmental carcinogens on buccal cells showed, that a high random variability on the observed chromosome instability after genotoxin exposure^34^. As many different cancer types were grouped in this study and confounding effects could not be excluded either, these non-significant observed changes may be result of the high variability sometimes observed in buccal cells.

Taken together, no increase of DNA damage in buccal cells in patients with neurodegenerative diseases compared to a reference cohort was observed. The buccal cell MNtest can be used to detect a variety of influencing factors. In particular, an association between altered MN frequency and cell death markers on one hand and reduced mental capacity on the other hand, was observed. Also, depression was found to be an influencing factor. Nevertheless, more data are needed in order to determine whether the buccal MNcytome assay is useful for predicting mental disorders. Confounding factors have to be considered during interpretation of test results and may influence the outcome of the assay.

## Materials and methods

### Recruitment of participants

The study was approved by the Ethical Advisory Board of the Medical Faculty of the University of Würzburg before the start of sample collection (No. 168/12) and performed in accordance with the relevant guidelines and regulations (i.e. Declaration of Helsinki). All individuals participated voluntarily, were informed about the study and informed consent was obtained from all participants. Two groups of patients were recruited in parallel. The first consists of 329 participants who were recruited during the second interview of a long-term cohort study^8^. None of them suffered from a severe internal, neurologic or psychiatric disease within the last year before sampling and this group is therefore referred to as reference group in the following. The second group consists of 96 individuals with a variety of different severe neurodegenerative diseases and was recruited from the psychiatric outpatient department of the University Hospital Würzburg, all were in the state to provide their own informed consent. This group is referred to as patient group.

### Sampling, preparation and staining

Sampling of buccal cells was conducted with cotton sticks, which were gently rubbed 10–15× along the inner side of each cheek. After that, the stick was placed in 4 ml 0.9% saline. The buccal cells were detached from the stick with vigorous movements. This procedure was repeated once. 10 ml of buccal cell buffer (0.01 M Tris–HCl, 0.1 M EDTA, 0.02 M NaCl, 1% penicillin/streptomycin solution, pH 7.0) was added and centrifuged for 5 min at 1000 rpm. Supernatant was discarded and 5 ml buccal cell buffer added before centrifugation. These steps were repeated twice and the remaining cells were resuspended in 1 ml. Via Cytospin centrifugation, slides with 8,000 cells each were prepared. Cells were fixed in methanol at − 20 °C for at least two hours and stained with 5 µl 1:100 Gel Green (Biotium) for 30 s. Evaluation of slides was conducted with a Nikon Eclipse 55i fluorescence microscope at 400× magnification.

### Scoring

The cells were scored according to the criteria previously described^17^. All mononuclear cells appeared to be differentiated and therefore all potentially basal or differentiated cells were counted as mononuclear cells in the following. Frequencies of binuclear cells, cells with condensed chromatin and karyolytic cells were counted as well as MN frequency. Only round nuclear anomalies inside the cytoplasm but outside the nucleus with similar staining to the nucleus but only 1/16 to 1/3 the size were counted as MN. Two slides with 1,000 cells each per individual were evaluated and averaged. MN were counted in mononuclear and binuclear cells as well as in cells with condensed chromatin.

### Statistics

Statistical evaluation was conducted with the software IBM SPSS Statistics 25 using the Kruskal–Wallis-Test for groupwise comparison and the two-tailed Mann–Whitney–U-Test for pairwise comparison. Statistical significance was assumed at *p* < 0.05.

## Supplementary Information

Supplementary Information.

## Data Availability

The datasets generated during the current study are available from the corresponding author on reasonable request.
