# Supplementary material for: Micronucleus frequency in buccal mucosa cells of patients with neurodegenerative diseases
Source: Sci Rep. 2020 Dec 17;10:22196. doi: 10.1038/s41598-020-78832-y (PMC7747561; doi:10.1038/s41598-020-78832-y)
Supplement: Supplementary file 1 — Supplementary Information. [file 41598_2020_78832_MOESM1_ESM.docx]

**Micronucleus frequency in buccal mucosa cells of patients with neurodegenerative diseases**

Hauke Reimann^1^, Helga Stopper^1^, Thomas Polak^2^, Martin Lauer^2^, Martin J. Herrmann^2^, Jürgen Deckert^2^, Henning Hintzsche(*)^1,3^

^1^ Institute of Pharmacology and Toxicology, University of Würzburg

^2^ Department of Psychiatry, Psychosomatics and Psychotherapy, Center of Mental Health, University Hospital Würzburg

^3^ Bavarian Health and Food Safety Authority, Erlangen

**Supplementary Figures**


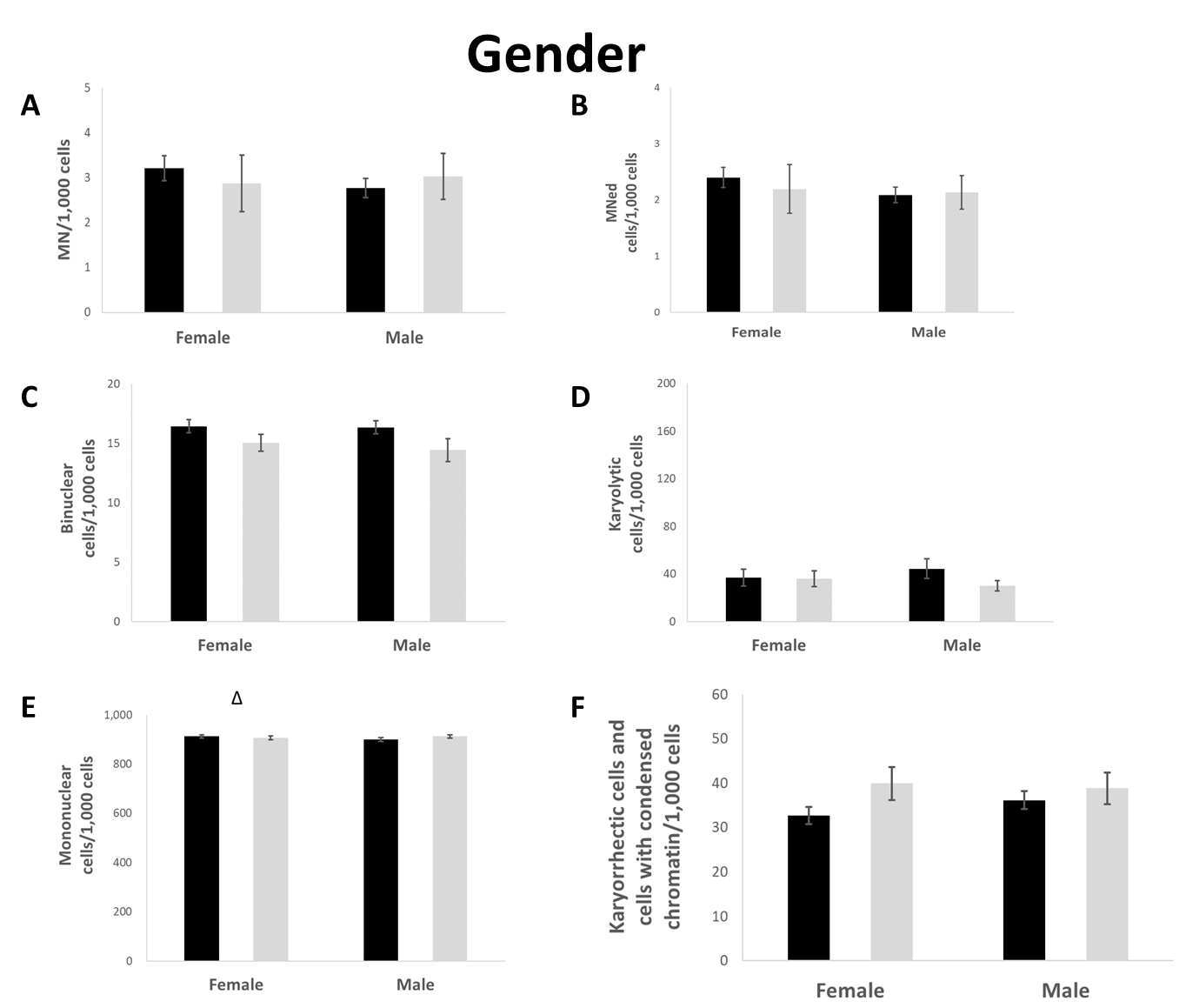


**Supplementary Fig. 1.** Rate of MN (A), MNed cells (B), binucleated cells (C), karyolytic (D), mononuclear (E) and karyorrhectic cells and cells with condensed chromatin (F) per 1,000 cells. Mean of each group ± standard error. Black bars represent data from reference, grey bars represent data from patients. n (female reference): 168; n (male reference): 161. n (female patients): 51; n (male patients): 44. Delta represent p-value < 0.05 after statistical evaluation referring to reference.


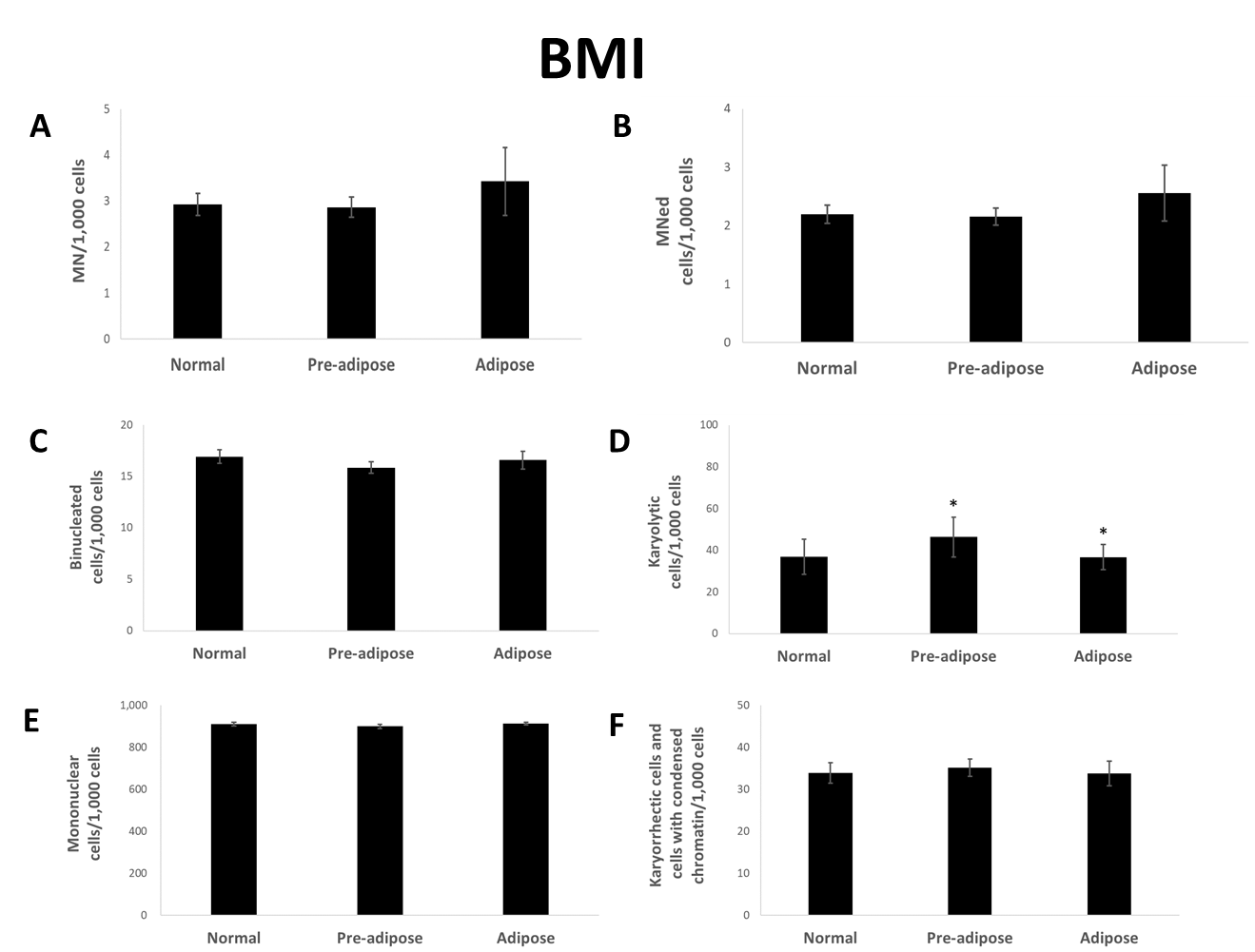


**Supplementary Fig. 2.** Rate of MN (A), MNed cells (B), binucleated cells (C), karyolytic (D), mononuclear (E) and karyorrhectic cells and cells with condensed chromatin (F) per 1,000 cells from reference distributed by BMI. Mean of each group ± standard error. n (normal): 134; n (pre-adipose): 142; n (adipose): 49. Asterisk represent p-value < 0.05 after statistical evaluation referring to normal weight group.
